# The haplotype-resolved autotetraploid genome assembly provides insights into the genomic evolution and fruit divergence in wax apple (*Syzygium samarangense* (Blume) Merr. and Perry)

**DOI:** 10.1093/hr/uhad214

**Published:** 2023-10-25

**Authors:** Xiuqing Wei, Min Chen, Xijuan Zhang, Yinghao Wang, Liang Li, Ling Xu, Huanhuan Wang, Mengwei Jiang, Caihui Wang, Lihui Zeng, Jiahui Xu

**Affiliations:** Fruit Research Institute, Fujian Academy of Agricultural Sciences, Fuzhou 350013, Fujian, China; Fujian Agriculture and Forestry University, Fuzhou 350002, Fujian, China; Shenzhen Branch, Guangdong Laboratory for Lingnan Modern Agriculture, Genome Analysis Laboratory of the Ministry of Agriculture, Agricultural Genomics Institute at Shenzhen, Chinese Academy of Agricultural Sciences, Shenzhen 518120, China; Fruit Research Institute, Fujian Academy of Agricultural Sciences, Fuzhou 350013, Fujian, China; Fujian Agriculture and Forestry University, Fuzhou 350002, Fujian, China; Fruit Research Institute, Fujian Academy of Agricultural Sciences, Fuzhou 350013, Fujian, China; Fruit Research Institute, Fujian Academy of Agricultural Sciences, Fuzhou 350013, Fujian, China; Fujian Agriculture and Forestry University, Fuzhou 350002, Fujian, China; Fujian Agriculture and Forestry University, Fuzhou 350002, Fujian, China; Fruit Research Institute, Fujian Academy of Agricultural Sciences, Fuzhou 350013, Fujian, China; Fujian Agriculture and Forestry University, Fuzhou 350002, Fujian, China; Fruit Research Institute, Fujian Academy of Agricultural Sciences, Fuzhou 350013, Fujian, China

## Abstract

Wax apple (*Syzygium samarangense*) is an economically important fruit crop with great potential value to human health because of its richness in antioxidant substances. Here, we present a haplotype-resolved autotetraploid genome assembly of the wax apple with a size of 1.59 Gb. Comparative genomic analysis revealed three rounds of whole-genome duplication (WGD) events, including two independent WGDs after WGT-γ*.* Resequencing analysis of 35 accessions partitioned these individuals into two distinct groups, including 28 landraces and seven cultivated species, and several genes subject to selective sweeps possibly contributed to fruit growth, including the *KRP1*-*like*, *IAA17-like*, *GME-like*, and *FLACCA-like* genes. Transcriptome analysis of three different varieties during flower and fruit development identified key genes related to fruit size, sugar content, and male sterility. We found that *AP2* also affected fruit size by regulating sepal development in wax apples. The expression of sugar transport-related genes (*SWEET*s and *SUT*s) was high in ‘ZY’, likely contributing to its high sugar content. Male sterility in ‘Tub’ was associated with tapetal abnormalities due to the decreased expression of *DYT1*, *TDF1*, and *AMS*, which affected early tapetum development. The chromosome-scale genome and large-scale transcriptome data presented in this study offer new valuable resources for biological research on *S. samarangense* and shed new light on fruit size control, sugar metabolism, and male sterility regulatory metabolism in wax apple.

## Introduction

Wax apple (*Syzygium samarangense* (Blume) Merr. and Perry), also known as Java apple and wax jambu, is a nonclimacteric tropical fruit tree from the *Myrtaceae* family and is native to the Malay Archipelago [1]. The *Myrtaceae* family is composed of approximately 80 genera and 3000 or more species [2]. According to a few studies of *Myrtaceae* genomes [3, 4], the phylogenetic position of this species remains uncertain. The *Myrtaceae* family has traditionally been divided into two main groups: fleshy-fruited and dry-fruited [2]. As one of the largest genera of fleshy fruits in *Myrtaceae*, *Syzygium* species exhibit complex genetic diversity [5]. The species of *Syzygium* include *Syzygium aqueum* (water apple, 2*n* = 44), *Syzygium cumini* (java plum, 2*n* = 66), and *S. samarangense* (wax apple, 2*n* = 33, 42, 44, 66, and 88) [2]. The available phylogenetic topology information based on chloroplast genomes is inconsistent with geographical and morphological classification to some degree [6]. Few *Syzygium* species genomes are available to provide clear genetic relationships. Accordingly, it is necessary to study the genome information of wax apple to construct a more reliable *Syzygium* species phylogenetic tree. The acquisition of long contigs from autopolyploid or highly heterozygous plants is the major obstacle to obtaining accurate genome information, which therefore remains a major challenge [7, 8].

Wax apple fruit, which is usually eaten fresh, is bell-shaped and narrow at the base, with four fleshy calyx lobes at the apex. Because of its strong flowering ability, wax apple can fruit in any given season under proper cultivation measures. The fruit has the characteristics of apple-like crispness, an aroma of roses, low-acidity flavor and richness in antioxidant compounds that are beneficial to human health, and it has therefore become a popular exotic fruit [9, 10]. According to statistics from relevant Chinese authorities, the total production of wax apple fruit in Taiwan and Hainan provinces was 89 800 tons in 2019, with great benefit for local farmers and the country’s economy (data from http://www.stats.gov.cn/). To meet the needs of consumers and enrich the diet with high-quality wax apples with a composition that guarantees high nutritional value, it is important to maintain a suitable sugar content and good size. Among annual crops, the *Fruit Weight 2.2* (*FW2.2*) and *Physalis Organ Size* (*POS*) genes have been shown to modulate fruit size by regulating cell division or expansion in tomato [11, 12], and *FRUITFULL* (*FUL*)-like MADS-box gene *CsFUL1* was shown to modulate cucumber fruit size elongation through auxin transportation [13]. However, genetic information related to fruit size regulation in perennial fruit trees is still unclear. In addition, there are low sugar and sour component contents in the fruit of most wax apple varieties. The regulatory mechanism of sugar and acid metabolism in wax apple are also unknown. Therefore, genome assembly and whole-genome re-sequencing are needed to further clarify the regulatory mechanisms related to fruit quality in wax apple.

Seedlessness is an important target trait in fruit breeding [14–16]. Consumers prefer wax apple with the seedless trait, which are largely selected from bud mutation of wild-types. It is a great challenge for breeders to obtain new seedless wax apple cultivars by cross-breeding, and no new cultivars have been bred for decades. There is still a lack of research on the genetic regulatory mechanisms of wax apple. The seedless trait caused by male sterility has been developed in grape, tomato, and citrus. In plants, male sterility refers to the inability to produce dehiscent anthers, viable male gametes, and functional pollen. Previous studies have confirmed that there are two major categories of male sterility. Male sterility resulting from the genes in both mitochondria and nuclei is identified as cytoplasmic male sterility (CMS); male sterility resulting from nuclear genes alone is known as genetic male sterility (GMS) [17]. For years, wax apple breeding efforts were hampered due to the complex genetic diversity of this species and the lack of genome information. Therefore, an accurate reference genome of wax apple is essential for understanding the mechanisms regulating fertility and for accelerating genomic selection breeding efforts.

In previous work, a superior clone, ‘Tub Ting Jiang’ (‘Tub’), with large and seedless fruit, high sweetness (total soluble solids, ‘TSS’, content of approximately 11.81%) and beautiful colour, was selected [18]. We also collected two special wax apple varieties, ‘DongKeng 3’ (‘DK’) and ‘ZiYu’ (‘ZY’). ‘DK’ is a rootstock variety with rich seeds in all of its fruits. The fruit of ‘ZY’ is bright red and small but highly sweet (TSS of approximately 14.56%) with 0 to 2 seeds inside. These varieties will be good materials for studying the genome information of wax apple. Through the study of the wax apple genome, we hope to accelerate the breeding process and produce more new cultivars that are larger, sweeter and more colourful.

In this study, we aim to sequence and assemble ‘Tub’, which is an autotetraploid wax apple variety, to fill existing genomic information gaps in wax apple. This genome was used to conduct a comparative genomic analysis to further insight into the functional and structural features of the *S. samarangense* genome. Furthermore, we identified key genes associated with fruit size, sugar content, and male sterility, which are important breeding traits of wax apple. This genome will provide a valuable resource for further molecular functional analyses and benefit the acceleration of wax apple breeding.

## Results

### Genome assembly and annotation

**Figure 1:**
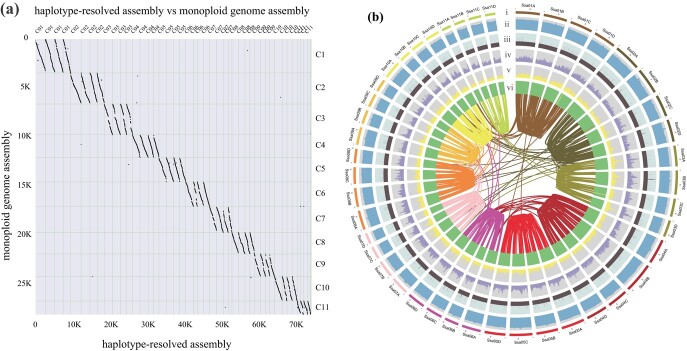
Alignment of the *Syzygium samarangense* monoploid genome with the *S. samarangense* genome and summary of the genome assembly. **(a)** A set of four homologous chromosomes aligned to a single monoploid chromosome. **(b)** From the outermost to innermost layer, the rings indicate the haplotype genomes in Mbp (**i**), GC content (**ii**), gene density (**iii**), SNP density (**iv**), SV density (**v**), expression (**iv**) and synteny blocks, respectively.

To investigate the features of the *S. samarangense* genome, we performed genome survey analysis, *K*-mer analysis, and 5S rDNA FISH experiment. The results verified that it was an autotetraploid genome with 44 chromosomes (2*n* = 4*x* = 44), and monoploid genome size was 420 Mb ((Figs SS1 andSS2, see online supplementary material). To generate a haplotype-resolved genome assembly, we sequenced a total of 92.0 Gb of PacBio subreads (~220 x of the estimated monoploid genome size), 90.0 Gb of Illumina short reads, and 92.40 Gb of high-throughput chromatin conformation capture (Hi-C) reads (Table SS1, see online supplementary material). A 1.49-Gb assembly with a contig N50 of 304.5 kb was assembled by using the CANU assembler [19] (**Table SS2**, see online supplementary material). All contigs were further anchored onto 44 pseudochromosomes with 11 homologous groups based on ALLHiC phasing. Finally, a total of 1.59 Gb of phased assembly sequences were obtained after gap filling, representing an allele-ware, chromosome-scale genome assembly with 98.9% completeness as evaluated by BUSCO (Fig. 1a; Fig. SS3, Table SS3, see online supplementary material) [20]. In addition, approximately 95.6% of the Illumina clean data could be aligned onto the genome assembly, covering 97.9% of the genomic regions (Table SS4, see online supplementary material), suggesting that high-quality genome sequences were acquired.

To obtain high-fidelity gene annotations, we applied two rounds of the MAKER pipeline to produce a set of 74 888 high-quality protein-coding gene models (Table 1). BUSCO analysis showed a completeness of 90.7%, with 69.3% duplication (**Table SS5**, see online supplementary material), indicating that the annotation included a mixture of genes and alleles. We adopted the pipeline developed in our previous project [21] to separate genes and alleles, resulting in a total of 24 016 genes with defined alleles. In addition, we annotated 952 tandemly duplicated genes and 11 161 dispersed duplicated paralogues (Table SS6, see online supplementary material).

**Table 1:** Statics of genome assembly in wax apple

Assembly feature	*Syzygium samarangense*
Total length of contigs (Gb)	1.49
Number of contigs	25 179
Contig N50 (Kbp)	304.5
Sequence anchored to chromosomes (Gb)	1.46
Anchor rate (%)	98
Monoploid genome assembly size (Gb)	0.41
Genes with defined alleles	24 016

The wax apple genome contained about 38.10% (593.25 Mb) of repetitive sequences. Long terminal retrotransposons (LTRs) were the predominant type of transposable elements (TEs) and accounted for 24.74% of the genome (**Table SS7**, see online supplementary material). The high proportion of LTRs was likely due to a recent large-scale burst that occurred ~0.1 million years ago (Mya) (Fig. SS4, see online supplementary material).

### Evolutionary history and whole-genome duplication

**Figure 2:**
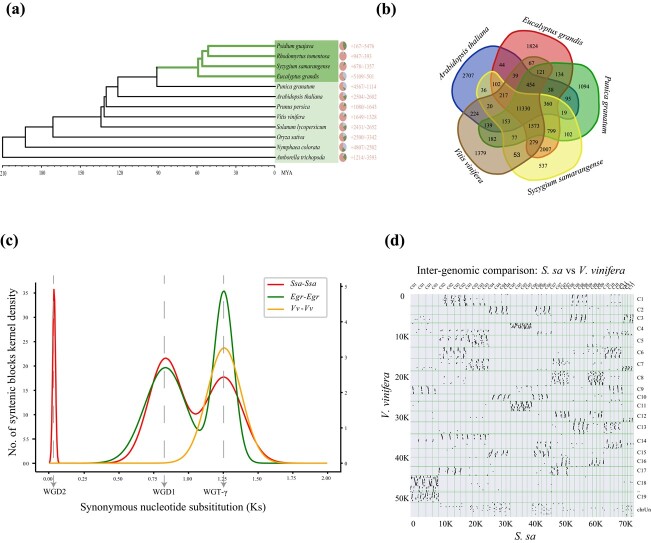
Phylogenetic and comparative analysis of *Syzygium samarangense*. **(a)** Phylogenetic tree of *S. samarangense*, *Eucalyptus grandis*, *Punica granatum*, *Arabidopsis thaliana*, *Vitis vinifera*, *Oryza sativa*, *Nyctophila colorata*, *Solanum lycopersicum*, *Psidium guajava*, *Prunus persica*, *Rhodomyrtus tomentosa*, and *Amborella trichopoda*. Gene family expansion/contraction analysis of the *S. samarangense* genome. The divergence times of *S. samarangense* and the other species are labelled at the bottom. **(b)** Orthologous and species-specific gene families in *S. samarangense* and the other species. **(c)** Distribution of synonymous substitution rates (Ks) among *S. samarangense* paralogues and orthologues with other species. **(d)** Alignment of the *S. samarangense* genome with the *V. vinifera* genome.

We identified 344 single-copy genes from 12 sequenced genomes by using OrthoFinder and subsequently employed them to construct a phylogenetic tree. The results clearly showed that *S. samarangense*, *Eucalyptus grandis*, *Psidium guajava*, *Rhodomyrtus tomentosa*, and *Punica granatum* belonged to the same branch of Myrtales. A significantly closer genetic relationship was observed between *S. samarangense* and *E. grandis*, which both belong to the *Myrtaceae* family. We further estimated divergence times and found that Myrtales arose 79.4 million years ago (Mya). Within the *Myrtaceae* family, *S. samarangense* and *E. grandis* diverged from each other 26 million years ago (Mya). According to a CAFE analysis, we characterized 1328 expanded gene families and 5363 contracted gene families (Fig. 2a). Gene Ontology (GO) enrichment analysis showed that the 1328 expanded gene families were primarily enriched in DNA polymerase activity, retrotransposon nucleocapsid, and mitochondrial fission. In contrast, the 5363 contracted gene families were primarily enriched in protein serine/threonine kinase activity, floral organ senescence, and secondary metabolite biosynthetic process (Figs SS5–S6, see online supplementary material). In comparison with other species, 537 unique gene families were identified (Fig. 2b) within the *S. samarangense* genome. These gene families were mainly enriched in a series of functional items, including catalytic activity, acting on DNA, retrotransfer, nucleocapsid, transfer, and RNA mediated (Fig. SS7, see online supplementary material).

Comparisons among the four haplotypes revealed 4.53 million SNPs, 0.49 million short indels, and 10 925 structural variations (SVs), and these genetic variations were evenly distributed along the 44 chromosomes (Fig. 1b; Table SS8, see online supplementary material). The clustering of chromosome-specific 13-mers partitioned each set of four haplotypes together (Fig. SS8, see online supplementary material), which was inconsistent with the allotetraploid *Miscanthus* genome and showed the separated distribution of subgenomes. The smudge plot analysis identified that the AAAB pattern was the dominant component, accounting for 56% of the examined *K*-mers (Fig. SS9, see online supplementary material). These results collectively indicated that *S. samarangense* has an autotetraploid genome with a high level of heterozygosity.

The distribution of synonymous substitutions per synonymous site (*K*_s_) of the homologous gene pairs clearly illustrated that the genome of *S. samarangense* experienced three rounds (WGT-γ, WGD-1, and WGD-2) of whole-genome duplication events (Fig. 2c). In addition to WGT-γ which is commonly found in the evolutionary history of grape and *E. grandis*, we discovered that *S. samarangense* and *E. grandis* had also undergone an independent whole-genome duplication (WGD-1). Compared with the situation in *E. grandis*, the specific WGD-1 event that appeared in the genome of *S. samarangense* was more complex. Moreover, the synteny relationship between *S. samarangense* and *Vitis vinifera* was further analysed to verify that WGD-1 and WGD-2 occurred after WGT-γ. As shown in Fig. 2d, the collinear relationship between *S. samarangense* and *V. vinifera* was 8:1, indicating that the occurrence of the two lineage-specific WGDs in *S. samarangense.*

### Genetic variations and population structure

**Figure 3:**
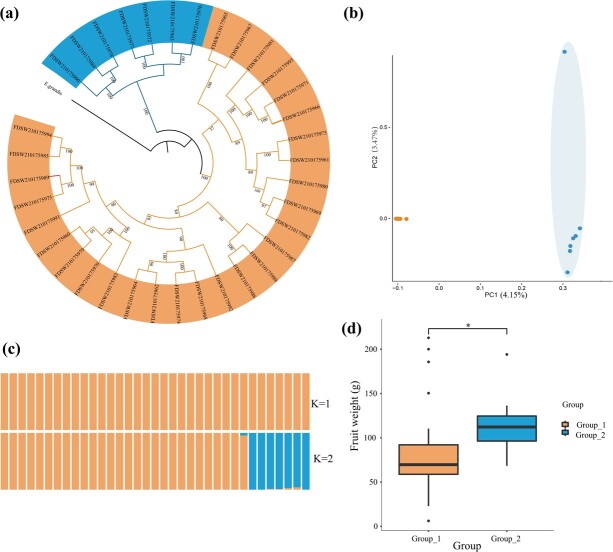
Phylogenetic splits and population genetic structure of 35 *Syzygium samarangense* accessions. **(a)** Maximum-likelihood tree of 35 resequenced *S. samarangense* individuals constructed based on 2 630 417 SNPs. **(b)** PCA plots of *S. samarangense* accessions showed two subgroups (Group_2, cultivars; Group_1, landraces). PC, principal component. **(c)** ADMIXTRUE analysis among the accessions revealed the distribution of K = 2 genetic clusters with the lowest cross-validation error. **(d)** Comparison of fruit weight between landraces and cultivars.

We resequenced 35 accessions of *S. samarangense* at the whole-genome level and identified 2 891 846 variants, including 2 630 417 SNPs and 261 429 indels (Table SS9, see online supplementary material). A total of 67 430 synonymous and 78 424 nonsynonymous mutations were identified (Table SS10, see online supplementary material). Phylogenetic analysis demonstrated that these *S. samarangense* were partitioned into two distinct groups. The commercially cultivated accessions were clustered together as the first group, and the remaining accessions, forming the second group, were landraces with limited or not artificial selection (Fig. 3a; Table SS11, see online supplementary material). The results of both principal component analysis (PCA) and genome structure analysis were consistent with phylogenetic analysis (Fig. 3b and c).

To identify the candidate genes that might have undergone natural or artificial selection during the evolutionary history of wax apple, we conducted a selective sweep-based SweeD analysis [22] in the 35 resequenced individuals*.* A total of 22.0 Mb of genomic sequences, covering 1299 and 1109 protein-coding genes, showed evidence of selective sweeps in the landraces and cultivars, respectively. These selectively swept regions were distributed along the 11 representative chromosomes that were selected from each set of homologous chromosomes, with some chromosomes having a higher density (Figs SS10–S11, see online supplementary material). GO enrichment analysis revealed that the swept genes in landraces were significantly enriched in the second-messenger-mediated signalling and calcium-mediated signalling pathways. However, the swept genes in cultivars were enriched in metabolic process and zygote asymmetric cell division (Figs SS12–S13, see online supplementary material).

Phenotypic analysis showed that the cultivated wax apples exhibited increased fruit weight compared with the landraces, leading to the hypothesis that fruit growth-related genes are likely under artificial selection (Fig. 3d). To verify this, we collected 30 homologous genes related to fruit growth in wax apple (Table SS12, see online supplementary material) based on the published genes in tomato [23]. We observed that the landraces contained three genes in the selectively swept genomic regions, namely *KRP1-like*, *IAA17-like*, and *GME-like*, which have been demonstrated to be involved in cell expansion, including endocycle control, auxin signalling, and ascorbate biosynthesis. In addition, the *FLACCA-like* gene, which is involved in ABA biosynthesis, was indicated to be under selection in cultivars [23] (Figs SS14–SS15, see online supplementary material).

### Genes contributing to fruit size and sugar content

**Figure 4:**
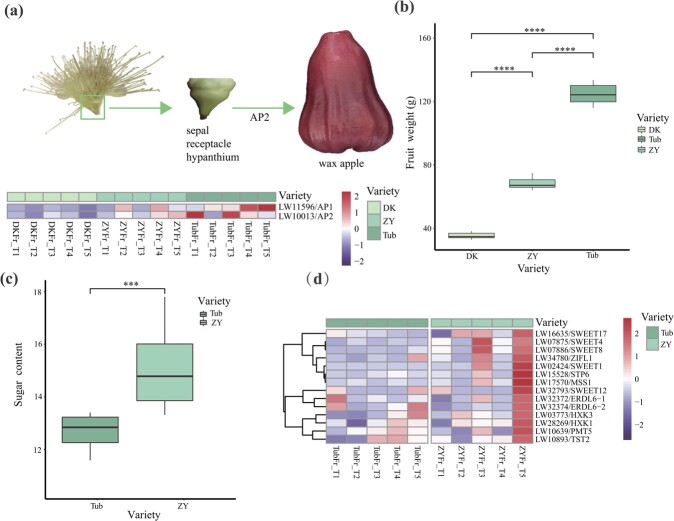
**.** Genes related to fruit growth and sugar content. **(a)** The expression of sepal development homologues (*AP1* and *AP2*) in ‘DK’, ‘ZY’, and ‘Tub’ during fruit development. **(b)** Comparison of fruit weight among ‘DK’, ‘ZY’, and ‘Tub’. ****P-value <0.0001, *t*-test, *n* = 10. **(c)** Comparison of sugar content between ‘Tub’ and ‘ZY’ fruit at maturity. ***P-value <0.001, *t*-test. **(d)** Expression of the candidate genes related to sugar transport (*SWEETs*, *ERDLs*, and *TST*) in the pink module in ‘DK’ and ‘Tub’ during fruit development. ‘DK’: ‘Dongkeng’; ‘Tub’: ‘Tub Ting Jiang’. FrT1, FrT2, FrT3, FrT4, and FrT5 represent 10 to 50 DAFB (days after full bloom) at approximately 10-day intervals.

We conducted fruit quality analysis on the fruits of all 35 *S. samarangense* materials. Based on the data of fruit size and TSS content we obtained (Table SS13, see online supplementary material), three representative accessions (‘Tub’, great size and medium sweetness; ‘ZY’, medium size and high sweetness; ‘DK’, little size and low sweetness) were selected for further studies (Fig. SS16, see online supplementary material). The ‘Tub’ variety exhibited the greatest fruit weight, with an average of 124.60 g per single fruit. This was almost two times than that of ‘ZY’ (68.47 g on average) and four times that of ‘DK’ (35.41 g on average), indicating that the fruit sizes of the three accessions were significantly different. A previous study indicated that the sepal development gene *APETALA* (*AP*) controls the fruit size in apples [24], which the fruit structure was similar to wax apples. Based on comparative RNA-Seq data, we found that the expression of *AP1* and *AP2* genes was highest in the ‘Tub’ accession, which had the greatest fruit weight, followed by the ‘ZY’ and ‘DK’ accessions, which had much reduced fruit sizes (Fig. 4; Figs SS17–S19 and Table SS14, see online supplementary material). The *AP1* gene was highly expressed in ‘Tub’Fr_T1 and ‘Tub’Fr_T3 samples, suggesting that *AP1* may play a role in promoting fruit growth at the early stage of fruit development.

In fruits, sugar content is usually defined as the TSS content that determines sweetness and is an important index in determining fruit quality. We observed that the fruits of ‘ZY’ presented a significantly higher soluble solid content than those of ‘Tub’ and ‘DK’ (14.56% vs. 11.81% vs. 4.67; Fig. 4c;Fig. SS16, see online supplementary material). We further queried the meaningful genes contributing to the elevated sugar content through a comparative RNA-seq analysis of fruit samples between ‘ZY’ and ‘Tub’. WGCNA identified 14 co-expressed modules (Fig. SS20, see online supplementary material), and a total of 400 genes were co-expressed in the ‘ZY’Fr_T5 sample, which is the most mature stage of ‘ZY’ fruit and presumably shows the highest sugar content (Fig. SS21, see online supplementary material). We observed that a list of important sugar transporter genes exhibited significantly high levels of expression in ‘ZY’Fr_T5 (Fig. 4d).

### Genes associated with male sterility

**Figure 5:**
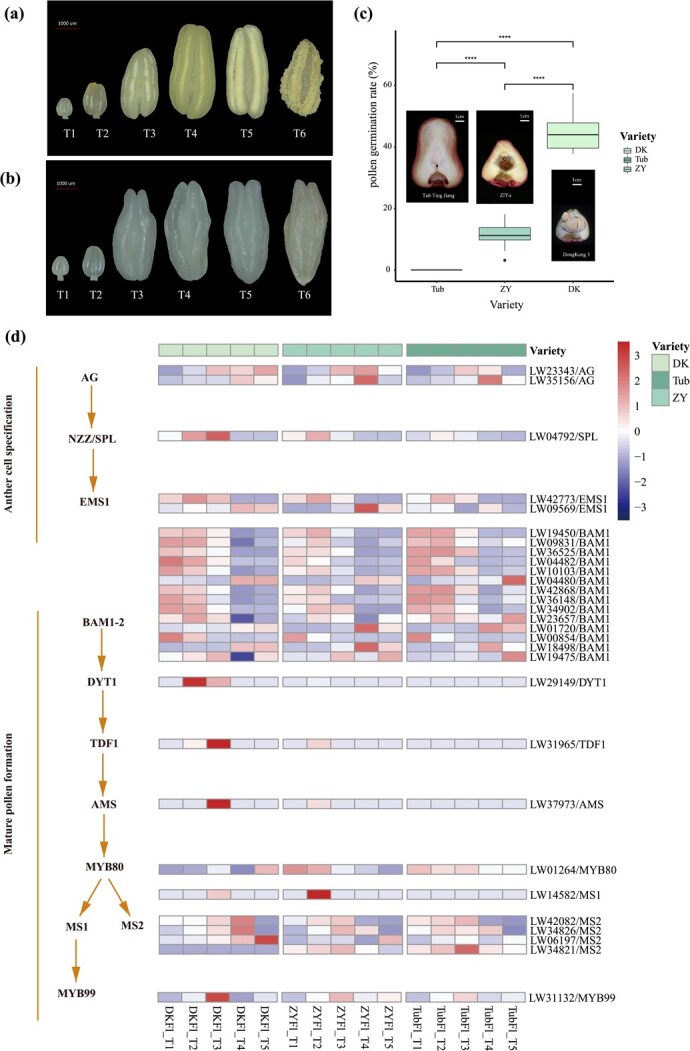
Anther development, pollen germination rate, and expression of anther and pollen development-related genes in ‘DK’, ‘ZY’, and ‘Tub’. **(a)** Anther development and dehiscence in ‘DK’. T1-T5 are consistent with FlT1-FlT5, and T6 represents 12 hours after blooming. **(b)** Anther development in ‘Tub’. T1-T5 are consistent with FlT1-FlT5, and T6 represents 12 hours after blooming. **(c)** Pollen germination rate of ‘Tub’, ‘ZY’, and ‘DK’. ****P-value <0.0001, *t*-test, *n* = 10. **(d)** Expression (FPKM) of anther and pollen development-related genes in ‘DK’, ‘ZY’, and ‘Tub’ from flowers at different stages, including FlT1, FlT2, FlT3, FlT4, and FlT5.

Seedless fruits are highly desirable due to their commercial value. This trait likely results from abnormal ovule and pollen development [25]. The results showed that the anther contained abundant pollen and exhibits normal dehiscence in ‘DK’, but the anther contained only a small amount of pollen and exhibited abnormal dehiscence in ‘Tub’ (**Fig. 5****a and b**). Subsequently, we determined the pollen germination rate in ‘DK’, ‘ZY’, and ‘Tub’. The results showed that the pollen germination rates were 11.73% and 45.06% for ‘ZY’ and ‘DK’, respectively, but the pollen of ‘Tub’ was not collected because of abnormal anther dehiscence (**Fig. 5c**).

In our study, samples from different flowering stages were used for further RNA-seq analysis to identify key genes involved in the development of pollen and anthers. WGCNA was performed to explore the potential genes related to male sterility in ‘Tub’. The coexpression network was constructed based on the correlation of gene expression in all samples. Finally, 16 different modules, defined as highly interconnected gene clusters, were identified and marked with different colours (Fig. SS22, see online supplementary material). Among these modules, three potential pollen and anther development-associated module eigengenes were characterized (Table SS15, see online supplementary material). In ‘DK’, the turquoise, tan, and darkgreen modules were correlated with the development of pollen and anthers (Figs SS23–S25, see online supplementary material). Interestingly, the turquoise module contained highly connected hub genes, including *LBD10*, *RPG1*, *RBOHE*, *CALS5*, *SK32*, and *MYB33*, which are known genes involved in pollen development (Table SS16 and Fig. SS26, see online supplementary material).

Furthermore, we identified a total of 29 homologous genes that played an important role in male sterility in *Arabidopsis*. These genes were mainly involved in anther cell specification and mature pollen formation pathways, and many of them showed differential expression at five different flower developmental stages (FlT1 to FlT5) among the three examined varieties (Fig. 5; Fig. SS27, see online supplementary material). The anther cell specification related gene nozzle/sporocyteless (*NZZ/SPL*) was found to be more highly expressed in ‘DK’ than in ‘Tub’. We also observed that dysfunctional tapetum 1 (*DYT1*), tapetum development and function 1 (*TDF1*), and abortive microspore (*AMS*) genes were specifically expressed in the FlT2 and FlT3 stages in ‘DK’, and barely expressed in ‘Tub’. In addition, the expression of three male sterile 2 (*MS2*) homologous genes in ‘DK’ was much higher than that in ‘Tub’ at the FlT4 and FlT5 stages. The expression pattern of these pollen development-related genes was consistent with the results of the pollen germination rate (Fig. 5d).

## Discussion

Wax apple is an economically important fruit crop that is widely cultivated throughout the Southeast Asian countries. Here, we generated a high-quality fully phased autotetraploid genome assembly and 35 resequencing accessions. These data represent comprehensive genomic resources for this species, facilitating the investigation of meaningful genetic variations and evolutionary history. Comparative genomics and transcriptomic analysis also revealed key genes underlying fruit growth, fruit size, and sugar content as well as factors related to male sterility caused by aborted pollen.

The assembly of the wax apple genome has been severely hindered by the high level of repetitive sequences and polyploidy. To date, only a few autotetraploid genomes have been assembled at the chromosome level, including those of sugarcane (*Saccharum spontaneum*) [21], cultivated alfalfa (*Medicago sativa L.*) [26], a potato cultivar (*Solanum tuberosum*, ‘Otava’) [27], and *Rehmannia glutinosa* [28]. Among these, only the sugarcane and cultivated alfalfa genomes were assembled by combining the developed sequencing technologies and chromosome phasing algorithm, whereas the developed sequencing technologies and pollen genome were used in the potato cultivar assembly. Here, we generated a haplotype-resolved chromosome-level genome of *S. samarangense* consisting of 44 allelic chromosomes by combining sequencing technologies and a chromosome phasing algorithm. The high percentage of assembled genome size relative to the monoploid estimate and anchor rate indicated a high-quality, allele-ware, and chromosome-scale genome assembly, benefiting downstream analysis and molecular breeding.

Fruit size and sugar content affect consumer preference. Emerging evidence shows that floral organ development-related genes participate in fruit development and play different roles among species, mainly depending on the type of floral organ that develops into fruit tissues [29]. Previous studies have shown that *AP2* governs seed yield [30] and floral development (especially sepal development [31, 32]) in *Arabidopsis* and can affect fruit growth in apples [24]. Intriguingly, *AP2* inhibits fruit size in *Arabidopsis* but promotes fruit size in apple [24]. In apple, miR172 inhibits the expression of *AP2*, and the overexpression of miR172 reduces fruit size, which indicates that miR172 plays a vital role in fruit size via *AP2* [24]. The highest expression of sepal development genes (*AP1* and *AP2*) according to our results was found in the ‘Tub’ group, which showed the greatest fruit weight, suggesting that *AP1*/*2* may play an important role in the regulation of wax apple fruit size. Considering that wax apple is recognized as false fruit that develops from the ovary and sepals, genes regulating sepal development are likely related to fruit size. *APETALA2* (*AP2*) governs sepal development, and *APETALA2* (*AP1*) acts downstream of *AP2* [33]. The main reason for this phenomenon is that unlike the fruits of apple and *S. samarangense*, which grow from hypanthium (and sepals, receptacle), the siliques of *Arabidopsis* develop from ovary tissues [34]. In apple, the overexpression of *MdERDL6*–1 improves glucose (Glc), fructose (Fru), and sucrose (Suc) concentrations in transgenic apple fruit and increases the expression of *TST1*/*TST2*, indicating that the sugar content in vacuoles is mediated by the coordinated action of *MdERDL6*–1 and *MdTST1/2* [35]. In our study, *ERDL6*–1 and *TST2* were mainly expressed in the ‘ZY’ variety, which has a higher sugar content, indicating that sugar accumulation in the ‘ZY’ variety could be attributed to the higher expression of *ERDL6*–1 and *TST2*. Through a comparative RNA-seq analysis of fruit samples for meaningful genes contributing to the elevated sugar content observed in ‘ZY’ and ‘Tub’, we identified 14 co-expressed modules, and a total of 400 genes were found to be co-expressed in the ‘ZY’Fr_T5 sample, representing the most mature stage of ‘ZY’ fruit, presumably containing the highest sugar content; these genes were significantly enriched in a series of molecular functions, particular in sugar transporter activity items. In addition, high expression levels were observed in ‘ZY’Fr_T5 for a list of important sugar transporter genes, including those encoding sucrose transporters (*SUTs*), monosaccharide transporters (*MSTs*), and sugars will eventually be exported transporters (*SWEETs*) and *TMT2*. Our results collectively indicated that these sugar transporter-related genes contributed to the elevated sugar content in the fruit of wax apple.

Seedless fruit occupies an important position in domestic and international markets. In *Arabidopsis*, the *LBD10* orthologue can interact with *LBD27* to form a heterodimer and plays an essential role in pollen development [36], strongly suggesting its potential role in the regulation of male sterility in wax apple, and many species have been developed, such as grape and citrus [37, 38]. Male sterility caused by aborted pollen is the main pathway targeted to cultivate seedless fruit. Based on this evidence, we speculate that the male sterility of ‘Tub’ is possibly attributed to functional defects in a few key genes, especially *DYT1*, *TDF1*, and *AMS*, affecting early tapetum development. In *Arabidopsis*, previous investigations showed that *DYT1*, *TDF1*, and *AMS* mutants all display a fully male sterile phenotype [39–41]. The *DYT1-TDF1-AMS-MS188* genetic network was suppressed upon the mutation of *Fatty Acid Export 1*, causing defective pollen formation [42]. Trace concentrations of imazethapyr (IM) significantly decrease the gene expression of *DYT1*, *TDF1*, and *AMS*, which affects anther and pollen biosynthesis in *Arabidopsis* [43]. Here, we revealed that *DYT1*, *TDF1*, and *AMS* were highly expressed in the male-fertile variety ‘DK’ but were expressed at lower levels in ‘Tub’ and especially the male sterile variety ‘ZY’. Therefore, these genes may play a potential role in the regulation of fertility in wax apple. Together, the results indicated that male sterility produces seedless fruit and may be caused by the decreased expression of *DYT1*, *TDF1*, and *AMS*. The results suggest that these genes could play important roles in seedless phenotype formation, and the relative expression levels of *LBD10*, *RPG1*, *RBOHE*, *CALS5*, *SK32*, and *MYB33* versus those of *DYT1*, *TDF1*, and *AMS* seem to be key factors in this process in wax apple.

## Conclusion

Here, a haplotype-resolved autotetraploid genome assembly of wax apple was generated, and comparative genomic analysis revealed that *S. samarangense* has experienced three different rounds of WGD events, including two independent WGDs after WGT-γ*.* Transcriptome analysis was used to identify genes related to fruit size, sugar content, and male sterility. Combined with fruit weight, fruit development characteristics, and transcriptome data analysis, the *AP1* and *AP2* genes may regulate fruit size by regulating sepal development. Sugar transport-related genes (*SWEETs* and *SUTs*) were found to be more highly expressed in varieties with a higher sugar content in ‘ZY’. The low expression of *DYT1*, *TDF1*, and *AMS* in ‘Tub’ may be the main reason for its sterility. Our results provide a foundation for further study of the regulatory mechanisms of fruit quality and male sterility and can be used in the molecular-assisted breeding of wax apple, especially for seedless traits.

## Materials and methods

### Illumina short-read sequencing and genome survey

We chose the ‘Tub’ accession for de novo genome sequencing and assembly. The plant materials were maintained by the Fujian Academy of Agricultural Sciences, and young leaves were collected from an individual tree planted in the wax apple GenBank Field for of the Fujian Academy of Agricultural Sciences, Fujian Province, China (Coordinates: 26°7′53″N; 119°20′6″E) under voucher number GPLWFJGSS0058. Genomic DNA was isolated from young leaves using the Qiagen Plant Genomic DNA Kit according to the manufacturer’s instructions. Then, the qualified DNA samples were randomly fragmented with a Covaris S-series Instrument, and Illumina PCR-free libraries with an insert size of 350 bp were constructed using the TruSeq Nano DNA HT Sample preparation Kit (Illumina, San Diego, California, USA). Finally, the constructed libraries were sequenced with 150-bp paired-end sequencing using Illumina HiSeq PE. Using Illumina short reads, the genome size, repeat contents, and heterozygosity rate of *S. samarangense* were estimated using jellyfish2.2.7 software [44].

### Genome sequencing

A combination of single-molecule real-time sequencing (SMRT), Illumina sequencing, and Hi-C sequencing with error correction was applied to assemble the complete genome sequence of *S. samarangense*. For SMRT, genomic DNA was disrupted randomly with 6 kb–20 kb fragments by using g-TUBE (Covaris, Woburn, Massachusetts, USA) and sequenced on the PacBio Sequel platform, generating 110 coverage. For Illumina sequencing, six libraries (300 bp) were constructed using the Illumina TruSeq Nano DNA Library Prep kit, and the libraries were sequenced on the Illumina Hi-Seq 2000 platform. For Hi-C sequencing, two Hi-C libraries were constructed using a standard procedure and sequenced using the Illumina HiSeq X Ten sequencer.

### Genome assembly

The contig-level assembly of the wax apple genome incorporated Illumina short reads and PacBio CLR subreads. The PacBio subreads were subjected to the whole pipeline of Canu assembler v1.9 [19], followed by polishing using the Pilon program [45] to increase assembly accuracy. To construct the haplotype-resolved genome assembly, we first mapped the Hi-C reads to the polished contig assembly using BWA MEM (-5SPM) and extracted the uniquely mapped paired reads. The resulting BAM files were applied to haplotype phasing and scaffolding using the ALLHiC pipeline [46]. In addition, the chimeric scaffolds were manually corrected based on the Hi-C signals in the juicebox. To fill the gaps, first, TGS GapCloser [47] software was used to fill the gaps in the wax apple genome with 30X ultralong ONT data. After filling the genome, the number of gaps was significantly reduced. Then, we used Merqury [48] software to check the gap-filled genome and found that some errors were introduced compared with the previous filling. To correct these errors, we extracted all gap sequences filled by TGS GapCloser and checked the QV quality value of each gap and the error rate of the corresponding sequence in the genome using Mercury software. Finally, we filled the correct GAP in the initial chromosomal level genome. The quality of chromosome-scale assembly was assessed using a Hi-C heatmap.

### Genome annotation

To annotate protein-coding genes, we followed the method described in a previous study [49]. Briefly, we integrated evidence from RNA-seq, orthologous proteins, and *ab initio* gene prediction by carrying out two rounds of the MAKER pipeline. In the first round of MAKER, Trinity was used for *de novo* assembly by using the RNA-seq data [50], and RSEM was applied to calculate transcript abundance [51]. After filtering the valid transcripts, the rest were imported into the PASA program, and the candidate proteins were trained by *ab initio* gene prediction [52]. In the second round of MAKER, the candidate proteins were retrained *ab initio*. Hisat2 and StringTie were used for reassembly [53, 54]. Finally, we selected the better annotation of the two rounds of annotation. BUSCO (v5) software was applied to calculate the degree of annotation complement. We used the same method reported for in an autopolyploid sugarcane genome to construct a monoploid genome, identify alleles, and analyse allelic variations [21].

### RNA library construction and sequencing

To improve the prediction of gene annotations, we performed RNA-seq using different tissues of *S. samarangense* including flesh, flower, leaf, ovary, root, and stem. All tissues were collected and subsequently frozen in liquid nitrogen. Total RNA was extracted with the RNAprep Pure Plant Plus Kit (Tiangen) following the manufacturer’s procedure. Transcriptome libraries were constructed using the NEBNext® Ultra™ RNA Library Prep Kit for Illumina (NEB, UK) according to the manufacturer’s instructions and sequenced with 150-bp paired-end sequencing using the Illumina NovaSeq 6000 (Illumina, USA) platform.

### Phylogenetic analysis and estimation of divergence times

To construct the phylogenetic tree, single-copy orthologous genes were defined by OrthoFinder v2.3.1 [55] from protein sequences of seven species (*E. grandis*, *P. granatum*, *Arabidopsis thaliana*, *V. vinifera*, *Oryza sativa*, *Nymphaea colorata*, and *Amborella trichopoda*). Thereafter, protein sequences aligned by MUSCLE [56] and GBLOCKS [57] were used to trim ambiguous parts of the alignment. A phylogenetic tree was constructed using RAxML [58] utilizing the JTT + I + G + F model and 1000 bootstrap analyses. The divergence times among these species were estimated by r8s [59]. Whether the gene families had undergone the expansion or contraction events in the eight sequenced species was evaluated using CAFE2.2 [60].

### Synteny and whole-genome duplication analysis

To investigate the whole-genome duplication (WGD) events in *S. samarangense*, synteny analysis of the *S. samarangense* and *V. vinifera* genomes was performed. The *V. vinifera* genome and annotation were downloaded from Phytozome (https://phytozome-next.jgi.doe.gov/). We applied the MCScan (python version) pipeline [61] following the suggested best workflow. The syntenic regions in the *S. samarangense* and *V. vinifera* genomes indicated that *S. samarangense* experienced two WGD events after WGT-γ.

To test the reliability of this result, the synonymous nucleotide substitutions at synonymous site (Ks) values were estimated for *S. samarangense*, *V. vinifera*, and *E. grandis* genomes by using the YN00 program in the WGDi package with the Nei-Gojobori approach [62]. Because the base substitution rate differs in the three species, the method applied by Jinpeng Wang [63] was used to correct the evolutionary rates of duplicated genes. After fit and merge operations, the Ks peaks caused by the same WGD event could be found in the same place.

### Resequencing and population analysis

A total of 35 accessions were resequenced, including 28 landraces and seven cultivars. All accessions were collected from the wax apple GenBank Field of the Fujian Academy of Agricultural Sciences, Fujian Province, China. Young leaves were collected from each accession and flash frozen in liquid nitrogen for DNA isolation. Genomic DNA from each sample was isolated, and paired-end reads were sequenced on the Illumina NovaSeq platform. The adaptors and low-quality reads were trimmed using Trimmomatic [64], and clean reads were aligned to the reference genome of *S. samarangense* using BWA with the default parameters [65]. We identified variants following the GATK [66] best practices pipeline. HaplotypeCaller and GenotypeCaller were used to call variants from all samples. Maximum-likelihood trees were constructed using VCF2Dis (https://github.com/BGI-shenzhen/VCF2Dis).

To infer the subgroups among the resequenced *S. samarangense* accessions, admixture [67] was used with different *k* values (from 1 to 3), the optimal value determined in this study was *k* = 2. PLINK1.9 and VCFtools [68] were used to perform PCA. Finally, we used SweeD [22] to identify complete selective sweeps in the *S. samarangense* genome with the default settings.

### Transcriptome sequencing and identification of coexpression modules

The fruits from three wax apple accessions, ‘ZY’, ‘Tub’, and ‘DK’, were sampled from 10 to 50 DAFB (days after full bloom) at approximately 10-day intervals, representing five developmental stages, namely T1 to T5. Total RNA was extracted from flowers and fruits using the RNAprep Pure Plant Plus Kit (Tiangen), and cDNA libraries were constructed and sequenced by the Illumina NovaSeq 6000 (Illumina, USA) platform. Subsequently, we evaluated read quality using FastQC software (http://www.bioinformatics.babraham.ac.uk/projects/fastqc), and removed sequencing adapters and low-quality bases using Trimmomatic [64]. The clean data were aligned to the *S. samarangense* genome using HISAT2 (v2.0.5) [69], and the fragments per kilobase per million mapped fragments (FPKM) value was calculated using StringTie (v.1.2.3) [70]. The R package weighted gene coexpression network analysis (WGCNA) was used to cluster genes with similar expression based on the FPKM data [71]. Genes with |MM| > 0.8 and |GS| > 0.2 were selected for further analysis, and the network was represented and displayed using Cytoscape (v3.6.0) [72]. Male sterility- and flower development-related genes were retrieved from *Arabidopsis* (https://www.arabidopsis.org/), and the homologues of *S. samarangense* were identified by a BLASTP search of these sequences against all *S. samarangense* protein sequences.

### Fruit quality analysis and pollen viability determination

For fruit weight analysis, the fruits of all 35 *S. samarangense* materials (including 28 landraces and seven cultivars) were collected. Ten fruits were randomly selected from three trees for each *S. samarangense* material. Fruit weight was measured with a QUINTIX213-1CN electronic balance (Sartorius, Germany). To determine the total soluble solids (TSS) content, 1 cm^3^ of tissue was taken from the upper, middle, and lower parts of each fruit sample. Then, the sample were mixed and homogenized thoroughly with a mortar and pestle. The supernatant of the homogenate was used for soluble solids content determinations with a hand-held Brix metre PAL-1 (ATAGO, Japan). To analyse pollen viability, the pollen tube germination rate was measured. At 35°C, pollen was cultured for 12 hours in medium with a sucrose concentration of 15%, boric acid concentration of 50 mg/L, and agar concentration of 1%. Then, optical microscopy was used to observe pollen tube germination. Three fields of vision were randomly selected, and the total number of pollen grains and the number of germinated pollen grains were counted at the same time. The germination rate was calculated. The standard for defining budding pollen was that the length of the pollen tube exceeded the diameter of the pollen.

For each experiment, the significance of between-group differences was analysed using *t*-test. All statistical analyses were performed using IBM SPSS software. *P*-values <0.001 were considered statistically significant.

## Supplementary Material

Web_Material_uhad214

## Data Availability

The raw sequencing reads used for *de novo* whole-genome assembly and the final genome and annotation files have been deposited in the Genome Warehouse in National Genomics Data Center, under accession number GWHDUEN00000000 and GWHDVFZ00000000 which are publicly accessible at https://ngdc.cncb.ac.cn/gwh. Raw resequencing data were uploaded to the National Genomics Data Center (NGDC, https://ngdc.cncb.ac.cn/), submission ID: WGS034963; BioProject access number: PRJCA011822; BioSample access number: SAMC1129200; GSA access number: CRA010157.
